# Supplementary material for: Stochastic palmitoylation of accessible cysteines in membrane proteins revealed by native mass spectrometry
Source: Nat Commun. 2017 Nov 3;8:1280. doi: 10.1038/s41467-017-01461-z (PMC5668376; doi:10.1038/s41467-017-01461-z)
Supplement: Supplementary file 1 — Supplementary Information [file 41467_2017_1461_MOESM1_ESM.pdf]

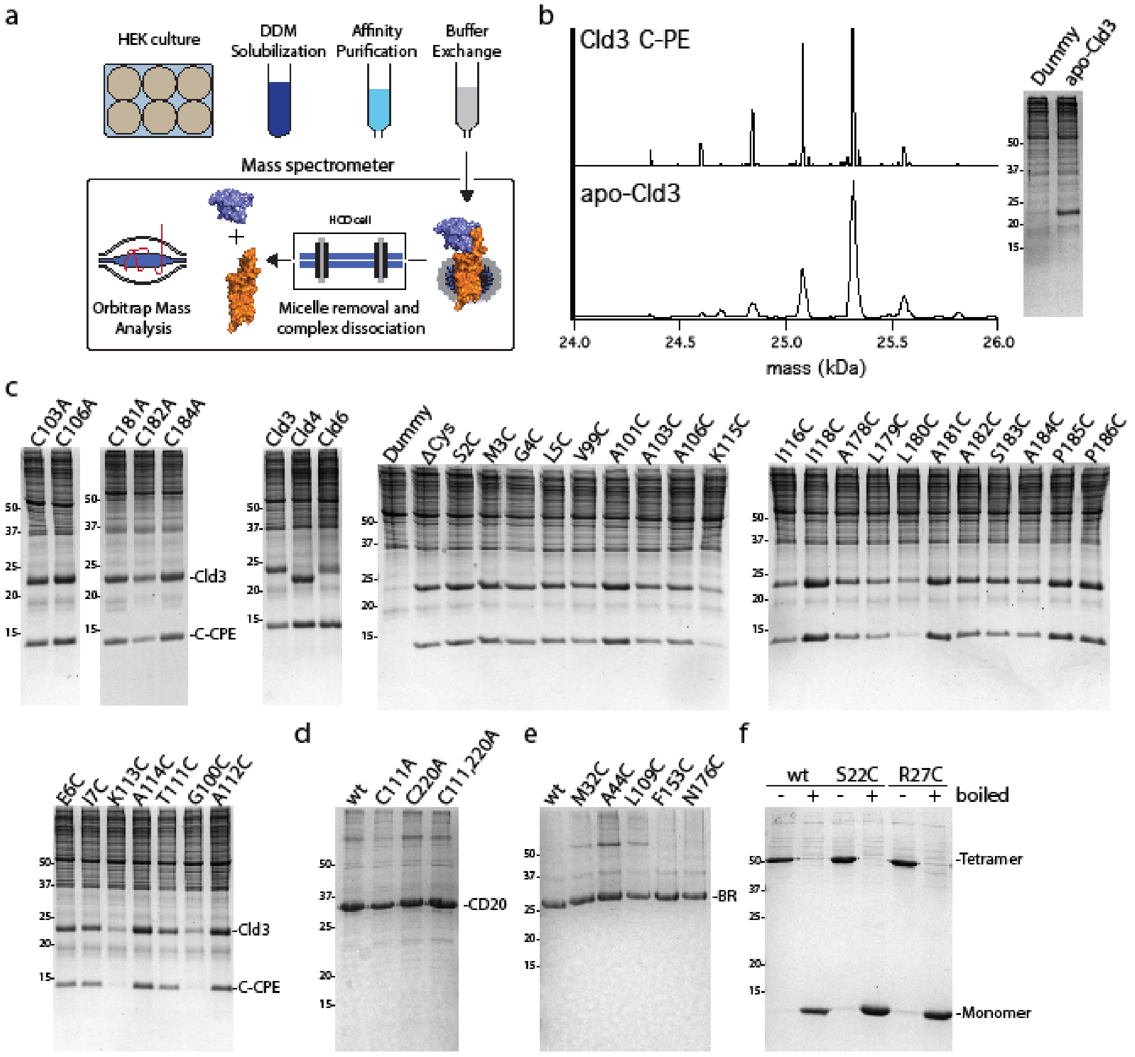


**Supplementary Figure 1.** **(a)** Workflow of the experiments described in this study. **(b)** Comparison of mass spectra of Cld3 purified with and without (apo-Cld3) C-CPE added to the culture medium. The apo-Cld3 spectrum was recorded at lower resolution resulting in peak broadening. **(c-f)** Representative SDS-PAGE gels of all Cld3 **(c)**, CD20 **(d)**, Bacteriorhodopsin (BR) **(e)** and KcsA **(f)** used in this study. Dummy represents purification from HEK293 cells transfected with dummy plasmid. All used KcsA constructs migrate according to a heat sensitive tetramer on SDS-PAGE, indicating all constructs are correctly folded.

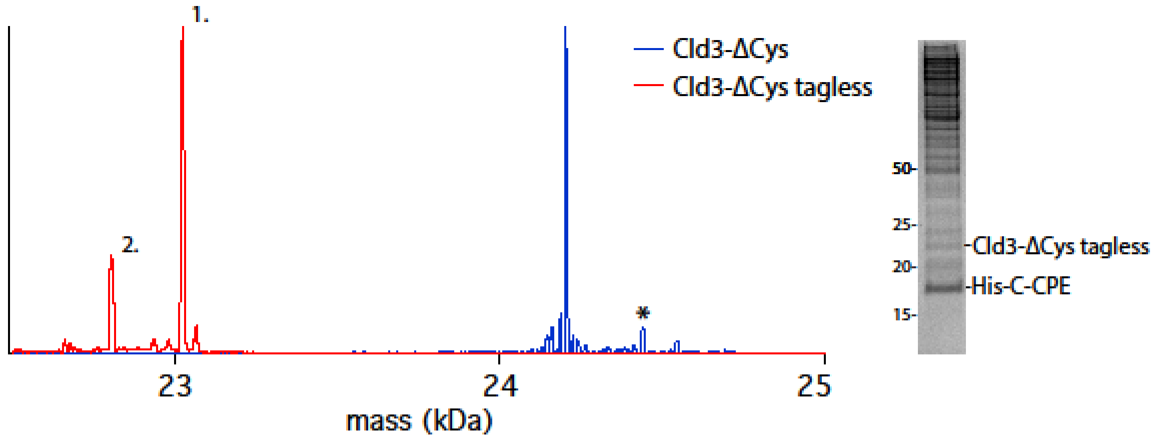


**Supplementary Figure 2.** Mass spectra of Cld3 with the putative palmitoylated Cys 103, 106, 181, 182 and 184 mutated to alanine (Cld3-ΔCys) and of Cld3-ΔCys with native N- and C- termini (Cld3-ΔCys tagless). The calculated masses of the two observed peaks for tagless Cld3-ΔCys correspond to full length protein (peak 1) and to Cld3 with transcription initiated at an alternative start codon at methionine 3 (peak 2). The peak corresponding to modification with an additional palmitate is annotated with an asterisk and is not observed in the Cld3-ΔCys with native termini. The SDS-PAGE indicates low purification yields of tagless Cld3-ΔCys. The Cld3-ΔCys tagless spectrum is representative of biological duplicates and Cld3-ΔCys is representative of biological triplicates.

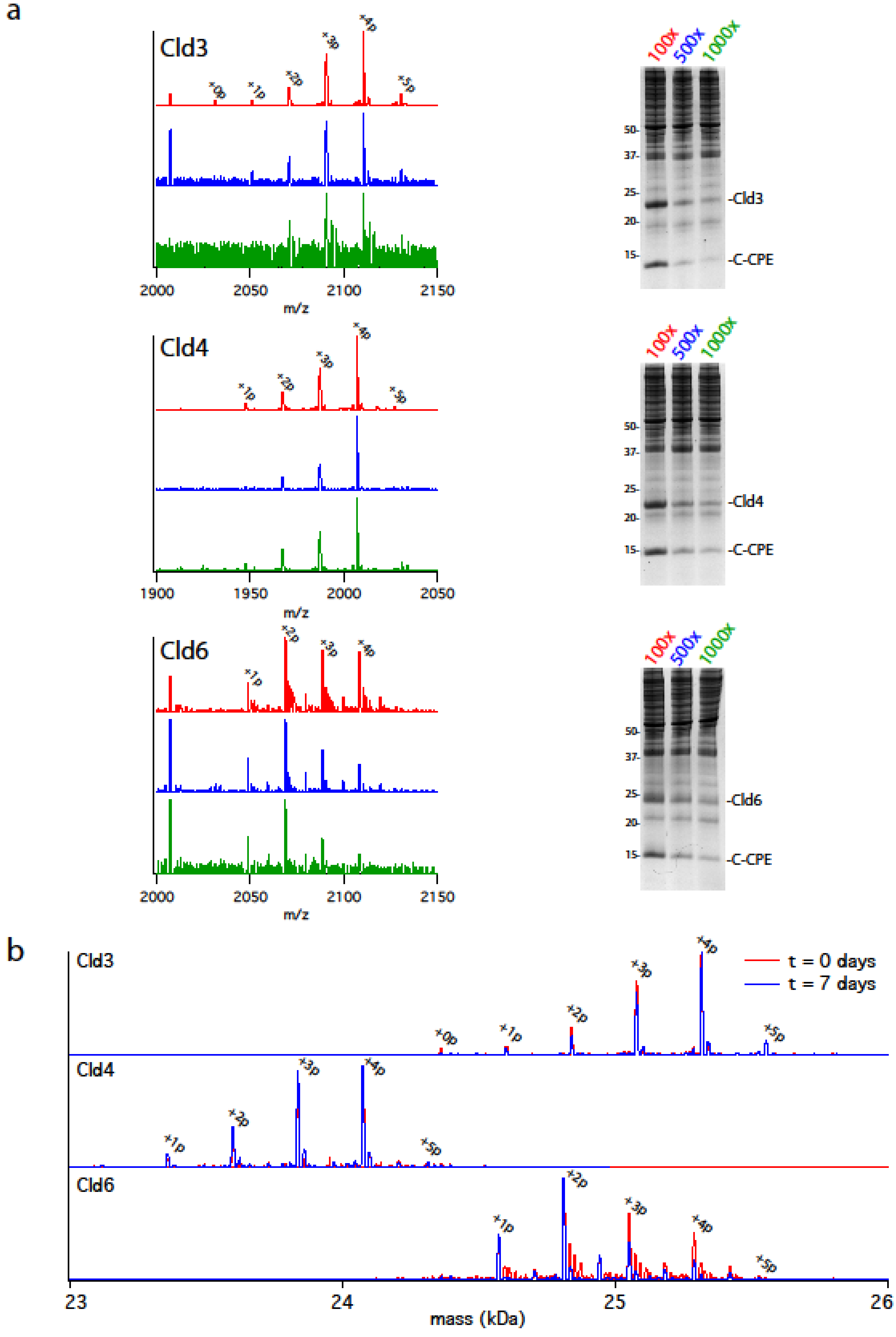


**Supplementary Figure 3. (a)** Palmitoylation levels of Cld3, 4 and 6 are

independent of protein-expression levels. Close up of the 12+ charge state of mass spectra obtained for claudins and claudin mutants with different expression levels, the spectra and gels are annotated with the dummy DNA dilution factor as described in the methods. Peaks are annotated with the number of attached palmitates. For each spectrum the corresponding SDS-PAGE gel is shown, the expression levels for 1000 x dilution are 3 fold reduced for Cld6, 5 fold for Cld3 and 6 fold for Cld6 as estimated by densitometry in Image Lab 3.0 (Bio-Rad) using the background band around 37 kDa as internal standard. **(b)** Deconvoluted mass spectra of Cld3, Cld4 and Cld6 after 7 days storage in detergent containing ammonium acetate buffer used for MS.

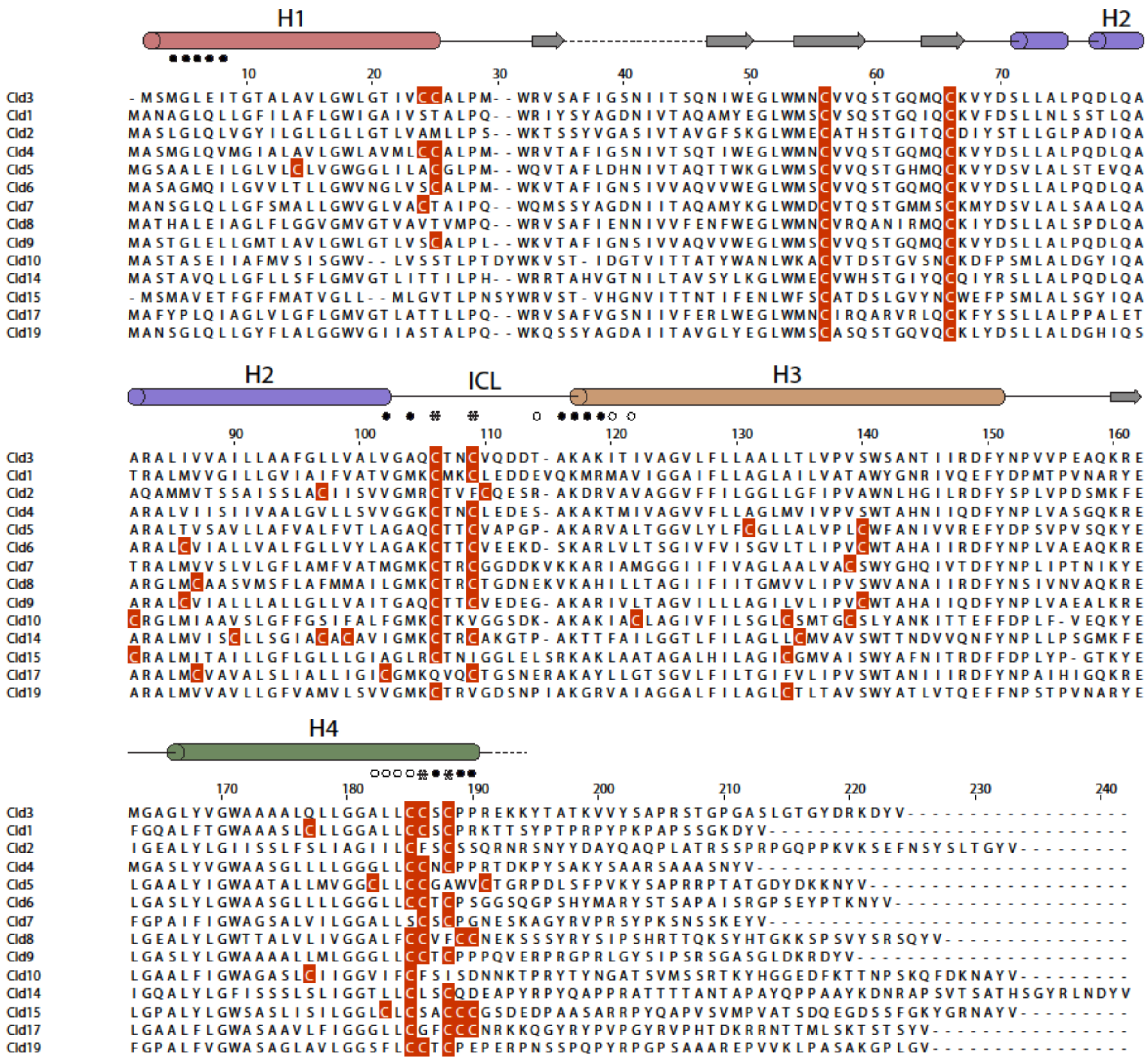

**Supplementary Figure 4.** Sequence alignment of classic human claudins. The secondary structure elements of the Cld3 homology model are indicated above the sequences as cylinders (α-helices), arrows (β-strands), lines (loops) and dashed lines (region not modeled due to lack of electron density in the crystal structure of the mCld15 template). Residues annotated with an asterisk are native Cld3 palmitoylation sites, a closed circle represents palmitoylated residues in cysteine-scanned Cld3 and an open circle represent unpalmitoylated residues in cysteine-scanned Cld3. The Uniprot IDs for the aligned sequences are: Cld3: O15551; Cld1:

O95832; Cld2: P57739; Cld4: O14493; Cld5: O00501; Cld6: P56747; Cld7: O95471; Cld8: P56748; Cld9: O95484; Cld10: P78369; Cld14: O95500; Cld15: P56746; Cld17: P56750; Cld19: Q8N6F1.

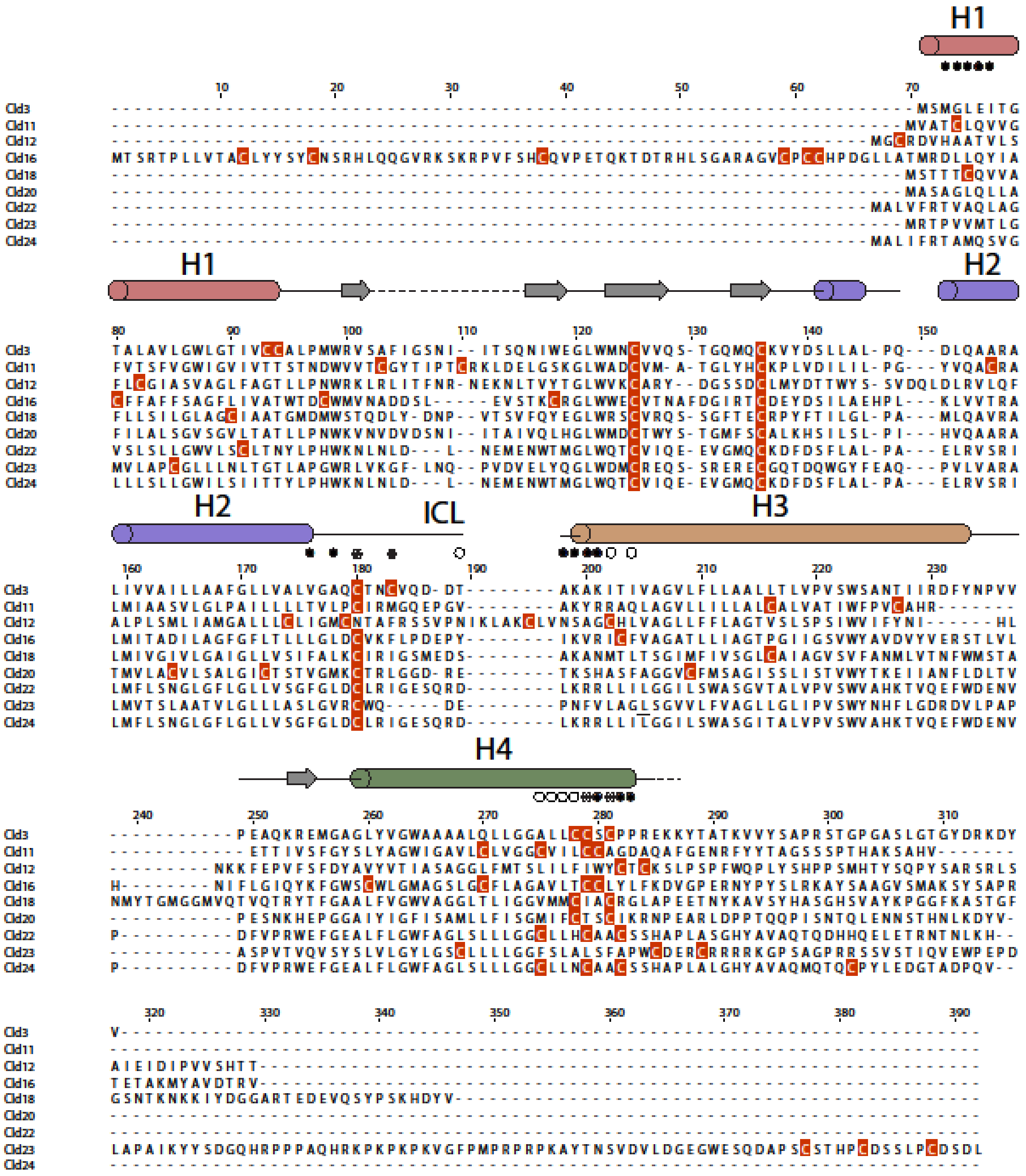

**Supplementary Figure 5.** Sequence alignment of all human claudins. Classic claudins except for Cld3 are not shown for clarity. The alignment is annotated as described in Suppl. Fig. 3. The Uniprot IDs for the aligned sequences are: Cld3: O15551; Cld11: O75508; Cld12: P56749; Cld16: Q9Y5I7; Cld18: P56856; Cld20: P56880; Cld22: Q8N7P3; Cld23: Q96B33; Cld24: A6NM45.

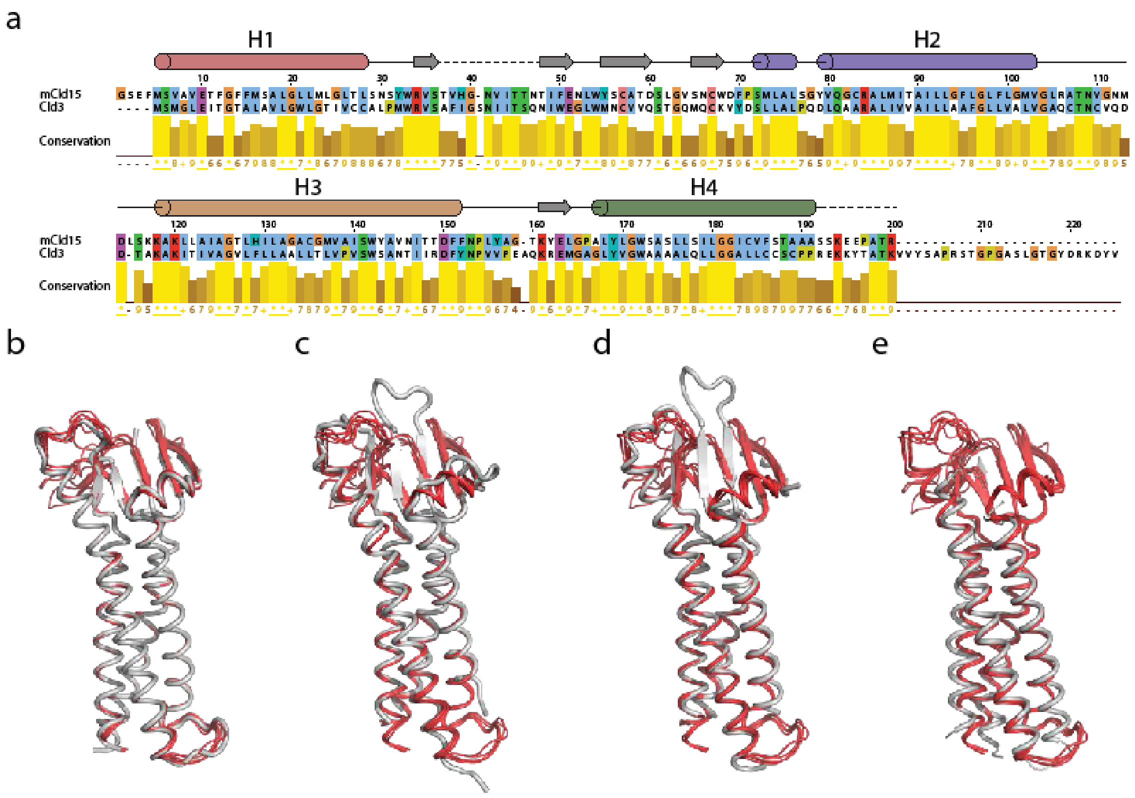


**Supplementary Figure 6.** Generation of a Cld3 homology model. **(a)** Sequence alignment of Cld3 (Uniprot ID: O15551) with the crystallized mCld15 construct[1]. The aligned regions are 38 % identical. **(b)** Overlay of the five generated homology models in red with mCld15 template in gray (PDB accession code 4P79), indicating the transmembrane helices are very similar. The average Cα atom RMSD of the template versus the generated models is 0.7 ± 0.09 Å. **(c)** Overlay of the generated homology models with Cld4 (PDB accession code 5B2G). The average Cα atom RMSD of Cld4[2] versus the generated models is 1.7 ± 0.07 Å and the sequence identity is 70 %. **(d)** Overlay of the generated homology models with mCld19[3] (PDB accession code 3X29). The average Cα atom RMSD of mCLD19 versus the generated models is 1.4 ± 0.07 Å and the sequence identity is 47 %. **(e)** Overlay of the generated homology models with rabbit voltage gated calcium channel $Ca_V1.1$ γ subunit[4] (PDB accession code 3JBR). The average Cα atom RMSD of the γ subunit versus the generated models is 1.6 ± 0.05 Å whereas sequence

identity is only 17 %, indicating that the claudin fold is very conserved. Structural alignments were made using secondary-structure matching[5] using residues 1 – 185 of the Cld3 models and sequence alignments and identity calculations were made using Clustal Omega[6].

a

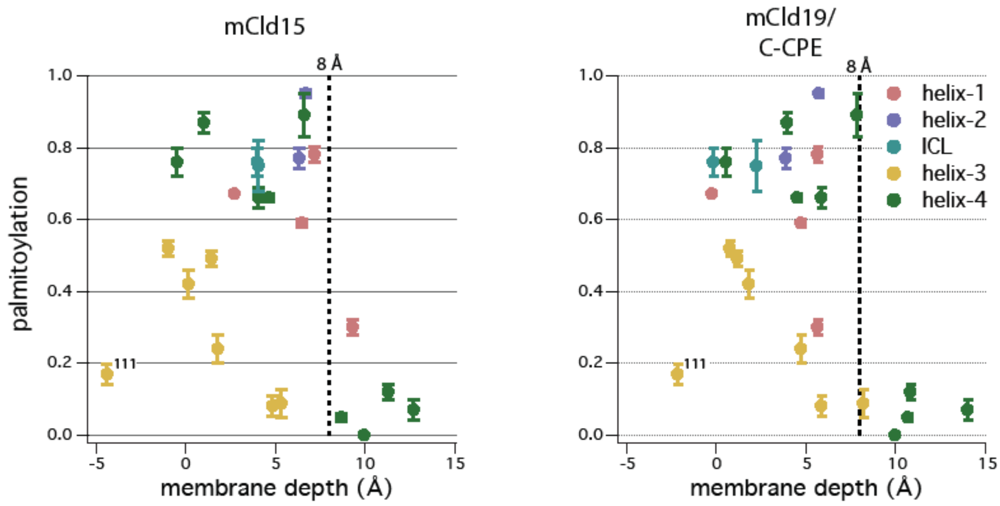


b

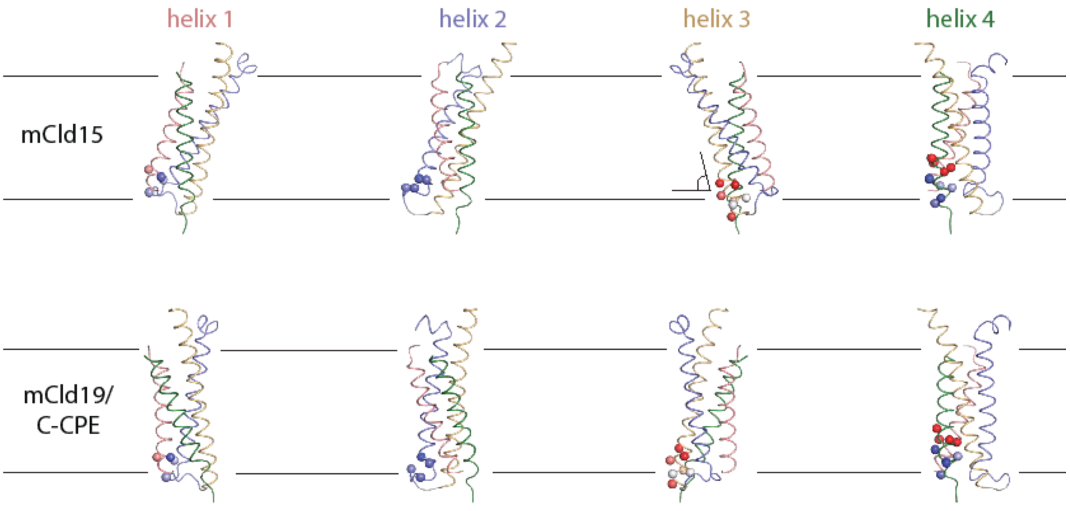


**Supplementary Figure 7.** The Cβ atom of all palmitoylated cysteines is located within 8 Å in the membrane. **(a)** Membrane depth of cysteine scanned residues, calculated using the membrane orientations of mCld15 and mCld19. No palmitoylation is observed on residues located up to 8 Å deep into the membrane in both orientations. Palmitoylation on residue 111 might be lost because it is located too far into the cytoplasm. Data are presented as mean values of biological triplicates and error bars represent standard deviations. **(b)** Membrane orientations of mCld15 as observed in the crystal structure and of mCld19/C-CPE

from the deposited simulation snapshot in memprotMD. The acute angle of mCld15 with the membrane at helix 3 is annotated. Spheres are colored as in Fig 2.

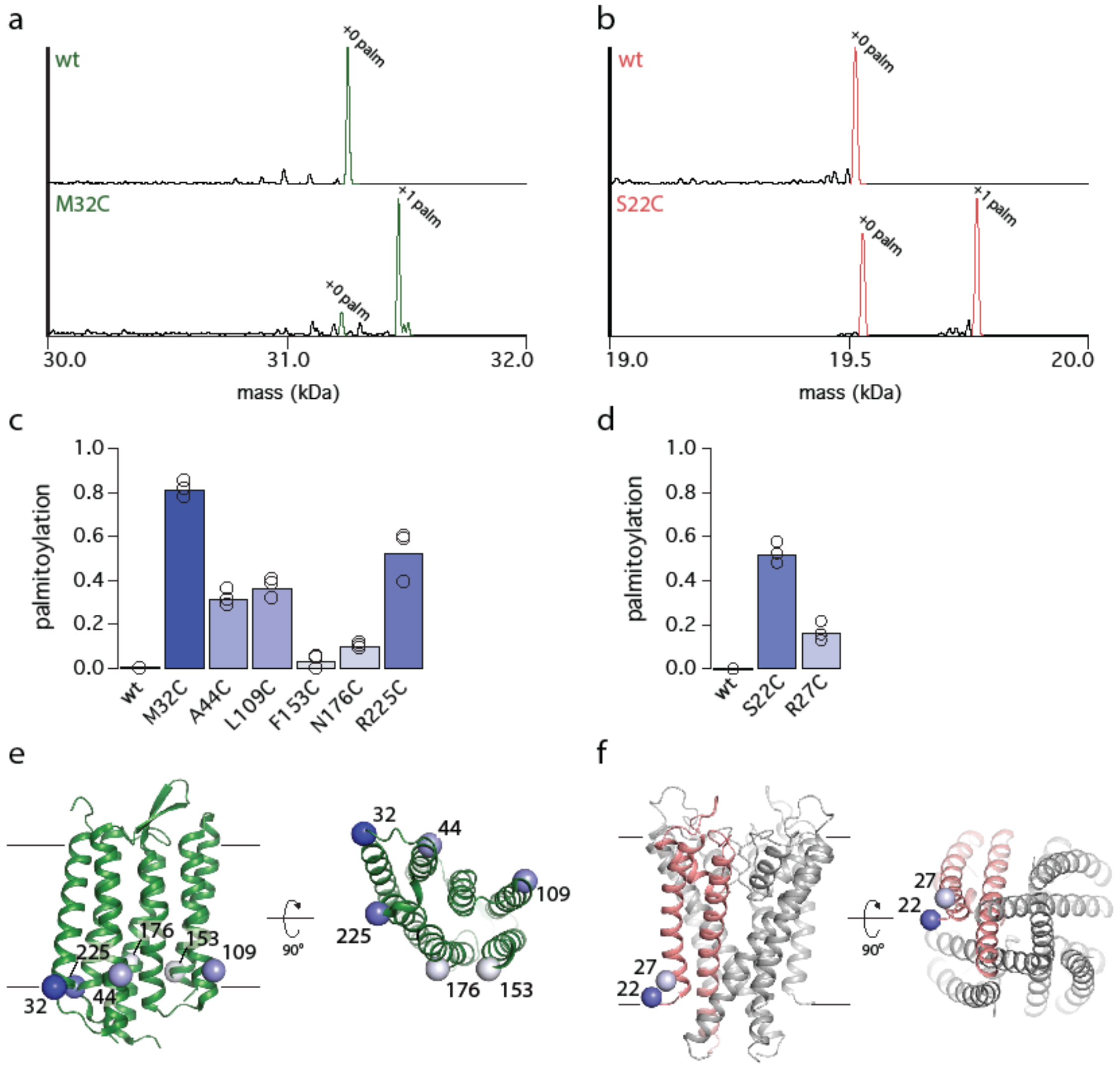


**Supplementary Figure 8.** Palmitoylation of heterologous expressed prokaryotic proteins bacteriorhodopsin and KcsA. **(a-b)** Representative spectra obtained for **(a)** native and mutant bacteriorhodopsin and **(b)** native and mutant KcsA. The mass of the annotated bacteriorhodopsin peaks corresponds to retinal bound bacteriorhodopsin with the zero or one palmitate bound. The mass of annotated KcsA peaks corresponds to monomeric KcsA with zero or one of palmitate bound. **(c-d)** Bar chart indicating the palmitoylated fraction of **(c)** bacteriorhodopsin and **(d)** KcsA mutants. Data are presented as mean values of biological triplicates and independent data points are shown as open circles. **(e)** Cartoon representation of bacteriorhosopsin crystal structure obtained with retinal bound, based on PDB

ascension code 1C3W[7]. Cα atoms of residues mutated to cysteines are shown as spheres and colored according to the observed palmitoylation in a white – blue gradient, in which full palmitoylation is shown as blue and no palmitoylation as white. **(f)** Cartoon representation of tetrameric KcsA based on PDB ascension code 1K4C[8]. Cα atoms of residues mutated to cysteines are shown as spheres and colored as described in **e**.

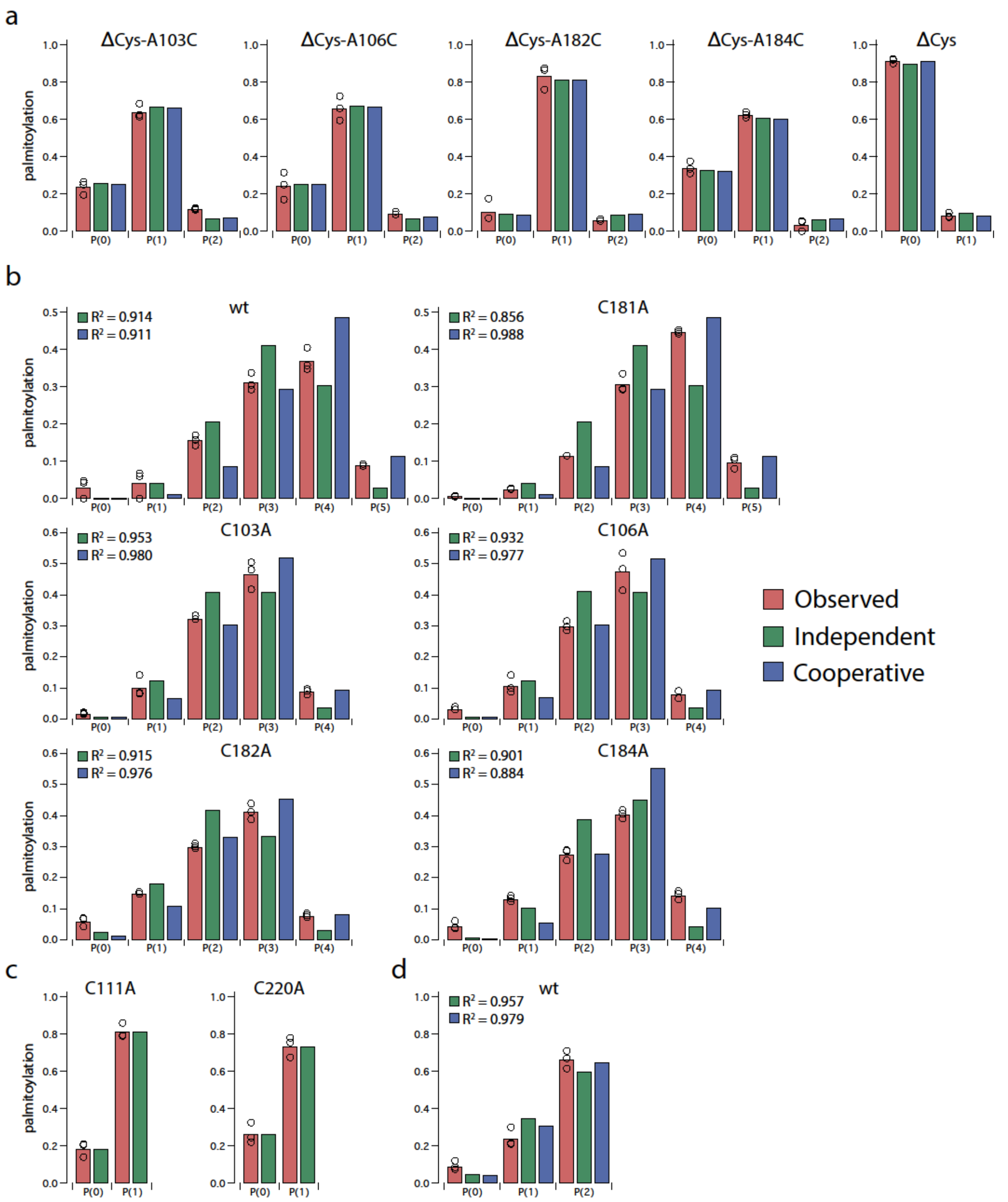


**Supplementary Figure 9.** Evaluation of stochastic independent and cooperative models in the quantitative analysis. **(a)** Comparison of the observed (red bars) and the fitted protein fractions for each palmitoylation state P(n) after fitting the stochastic model of independent events without (green) and with cooperativity (blue) to Cld3 mutants with a single cysteine at a native palmitoylation site and to Cld3 with all putative palmitoylated cysteines mutated to alanine (ΔCys). The

Cld3-ΔCys experiment shows a rudimentary level of palmitoylation, see main text. Both models fit the data equally well ($R^2$ = 0.993). All measured data in this figure are presented as mean values of biological triplicates and independent data points are shown as open circles. **(b)** Observed and predicted protein fractions of Cld3 with either four (wild type) or three cysteines at native palmitoylation sites. Predicted fractions are calculated with the independent (overall $R^2$ = 0.91) and cooperative model (overall $R^2$ = 0.96) using the probabilities and, if applicable, the cooperative parameter obtained from fitting the models to Cld3-ΔCys or Cld3 mutants with a single cysteine at native sites in **a**. **(c)** Observed and calculated protein fractions after fitting the independent model to CD20 mutants with a single cysteine at a native palmitoylation site. The model fits the data with $R^2$ = 0.99. **(d)** Observed and predicted protein fractions of wild type CD20 with two sites available for palmitoylation. Predicted protein fractions are calculated by the independent (overall $R^2$ = 0.96) and cooperative models (overall $R^2$ = 0.98) using the probabilities obtained from fitting the models to CD20 single cysteine mutants in **c**. The cooperativity parameter obtained from fitting the cooperative model to all Cld3 data was used for prediction by the cooperative model.

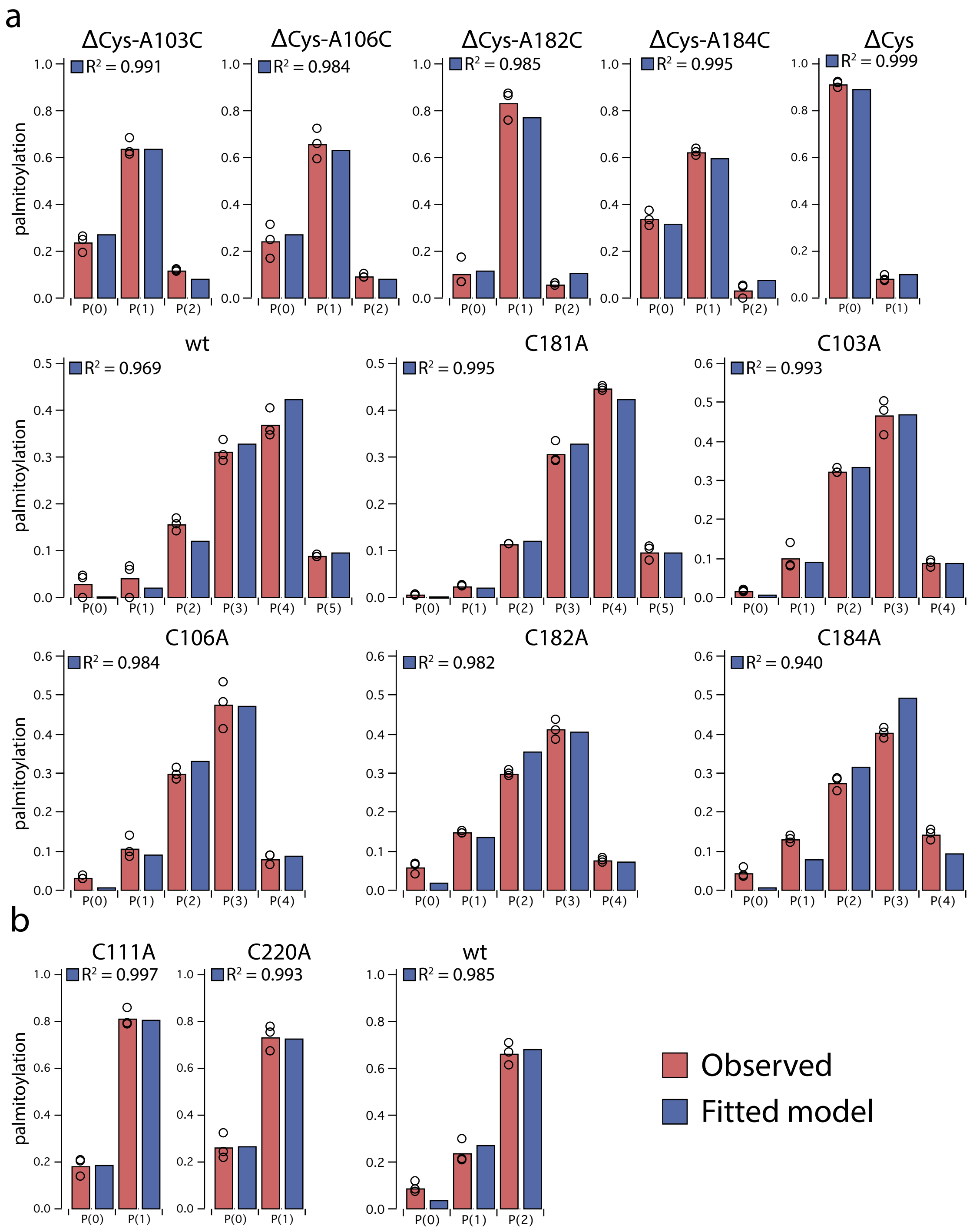


**Supplementary Figure 10.** Quantitative fit of all parameters of the cooperative model to all observed Cld3 and CD20 data. **(a)** Comparison of the measured and the calculated protein fractions obtained by fitting the cooperative model to Cld3 variants with one, three or four cysteines at native palmitoylation sites and to Cld3 with all putative palmitoylated cysteines mutated to alanine (ΔCys, overall $R^2$ =

0.99). **(b)** Measured and calculated protein fractions obtained by fitting the cooperative model to CD20 variants with one or two cysteines at a native palmitoylation site (overall $R^2$ = 0.99).

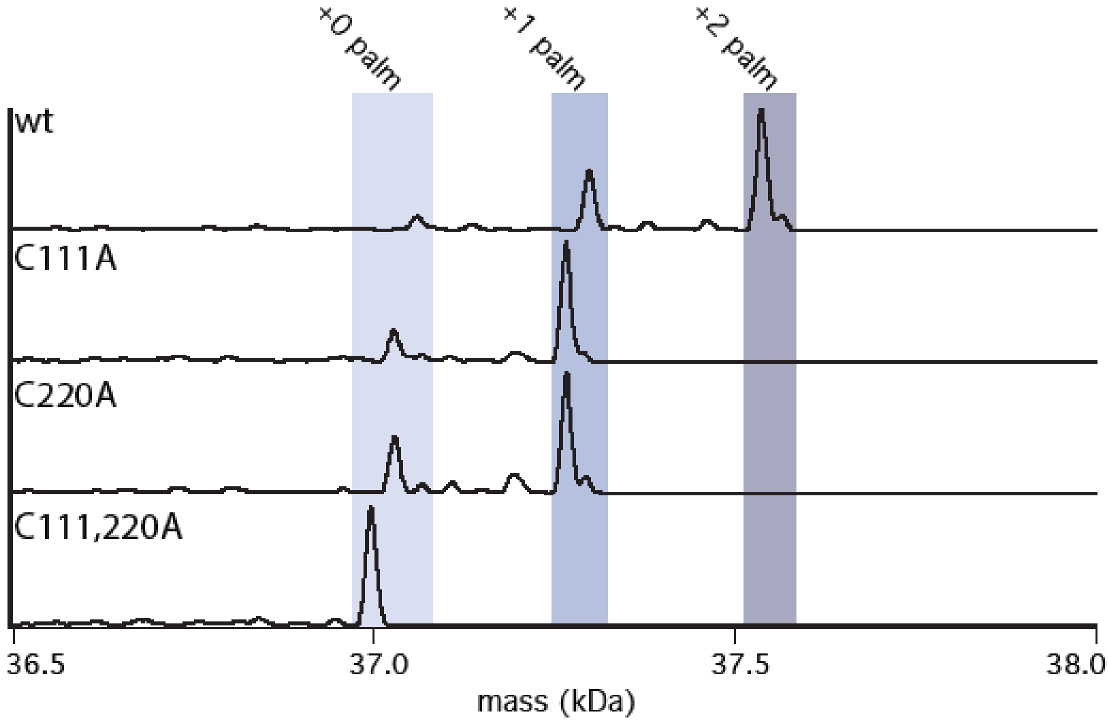


**Supplementary Figure 11.** Spectra obtained for wild type CD20 containing two palmitoylation sites and CD20 mutants containing either one or no palmitoylation sites. All panels in this figure show representative data from biological triplicates.

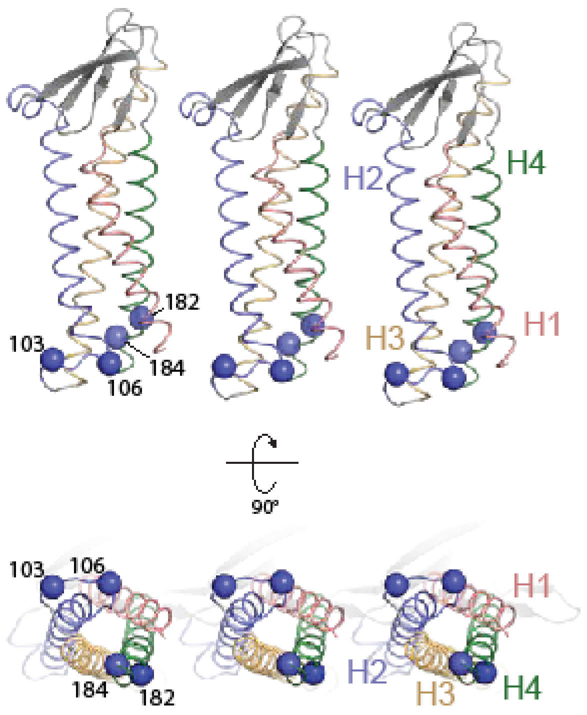


**Supplementary Figure 12.** Palmitoylation sites map to the flank of the proposed tight junction arrangement. The Cld3 homology model was superimposed on the observed tight junction arrangement in mCld15 crystals and the Cα atoms of native palmitoylated cysteines in Cld3 are shown as spheres and colored as in Fig. 4a.

| sample | # palmitoyl chains | experimental mass (Da) | | theoretical mass (Da) | difference (Da) |
|---|---|---|---|---|---|
| **Claudins** | | **avg** | **stdev** | | |
| Cld3 | 0 | 24361.9 | 0.25 | 24365.5 | -3.6 |
| | 1 | 24601.5 | 0.70 | 24603.9 | -2.4 |
| | 2 | 24840.5 | 0.31 | 24842.3 | -1.8 |
| | 3 | 25079.5 | 0.07 | 25080.7 | -1.2 |
| | 4 | 25317.8 | 0.12 | 25319.1 | -1.3 |
| | 5 | 25556.1 | 0.37 | 25557.5 | -1.4 |
| Cld4 | 0 | 23123.2 | 1.20 | 23124.2 | -1.0 |
| | 1 | 23359.5 | 0.85 | 23362.6 | -3.1 |
| | 2 | 23599.1 | 0.26 | 23601.0 | -1.9 |
| | 3 | 23838.1 | 0.11 | 23839.4 | -1.3 |
| | 4 | 24076.2 | 0.50 | 24077.8 | -1.7 |
| | 5 | 24313.6 | 0.72 | 24316.2 | -2.7 |
| Cld6 | 0 | 24335.3 | 2.07 | 24338.5 | -3.2 |
| with M1 | 1 | 24574.3 | 0.84 | 24576.9 | -2.6 |
| | 2 | 24813.1 | 0.19 | 24815.3 | -2.2 |
| | 3 | 25052.5 | 0.29 | 25053.7 | -1.2 |
| | 4 | 25291.2 | 0.23 | 25292.1 | -1.0 |
| | 5 | - | - | 25530.5 | - |
| without M1 | 0 | 24466.1 | 0.11 | 24469.7 | -3.6 |
| | 1 | 24705.0 | 0.86 | 24708.1 | -3.1 |
| | 2 | 24943.5 | 2.04 | 24946.5 | -3.0 |
| | 3 | 25183.1 | 0.49 | 25184.9 | -1.8 |
| | 4 | 25422.3 | 0.29 | 25423.3 | -1.0 |
| | 5 | - | - | 25661.7 | - |
| **native site mutants in Cld3** | | | | | |
| C103A | 0 | 24332.5 | 0.71 | 24333.4 | -0.9 |
| | 1 | 24570.6 | 0.40 | 24571.8 | -1.2 |
| | 2 | 24809.1 | 0.20 | 24810.2 | -1.1 |
| | 3 | 25047.6 | 0.12 | 25048.6 | -1.1 |
| | 4 | 25285.5 | 0.42 | 25287.0 | -1.6 |
| | 5 | - | - | 25525.4 | - |
| C106A | 0 | 24332.2 | 0.70 | 24333.4 | -1.2 |
| | 1 | 24570.8 | 0.63 | 24571.8 | -1.0 |
| | 2 | 24809.2 | 0.31 | 24810.2 | -1.0 |
| | 3 | 25047.4 | 0.15 | 25048.6 | -1.3 |
| | 4 | 25285.4 | 0.59 | 25287.0 | -1.6 |
| | 5 | - | - | 25525.4 | - |
| C181A | 0 | 24329.6 | 0.70 | 24333.4 | -3.8 |
| | 1 | 24568.9 | 0.27 | 24571.8 | -2.9 |
| | 2 | 24808.4 | 0.27 | 24810.2 | -1.8 |
| | 3 | 25047.2 | 0.19 | 25048.6 | -1.4 |
| | 4 | 25285.9 | 0.11 | 25287.0 | -1.2 |
| | 5 | 25524.3 | 0.34 | 25525.4 | -1.2 |
| C183A | 0 | 24330.3 | 0.69 | 24333.4 | -3.1 |
| | 1 | 24570.0 | 0.18 | 24571.8 | -1.8 |
| | 2 | 24809.1 | 0.07 | 24810.2 | -1.1 |
| | 3 | 25047.5 | 0.09 | 25048.6 | -1.1 |
| | 4 | 25285.8 | 0.57 | 25287.0 | -1.3 |
| | 5 | - | - | 25525.4 | - |
| C184A | 0 | 24330.2 | 0.30 | 24333.4 | -3.2 |
| | 1 | 24569.3 | 0.24 | 24571.8 | -2.5 |

| | | | | | |
|---|---|---|---|---|---|
| | 2 | 24809.2 | 0.16 | 24810.2 | -1.0 |
| | 3 | 25047.6 | 0.10 | 25048.6 | -1.0 |
| | 4 | 25285.6 | 0.30 | 25287.0 | -1.4 |
| | 5 | - | - | 25525.4 | - |
| **Cld3 cysteine scanning** | | | | | |
| 5CA | 0 | 24203.7 | 0.27 | 24205.2 | -1.5 |
| | 1 | 24443.7 | 1.14 | 24443.6 | 0.1 |
| 5CA – native termini | 0 | 23024.8 | 0.48 | 23025.0 | -0.2 |
| Alternative start codon | 0 | 22805.5 | 0.56 | 22806.8 | -1.3 |
| M3C | 0 | 24176.1 | 0.06 | 24177.1 | -1.0 |
| | 1 | 24414.3 | 0.22 | 24415.5 | -1.2 |
| | 2 | 24652.5 | 0.38 | 24653.9 | -1.4 |
| G4C | 0 | 24251.3 | 1.07 | 24251.2 | 0.1 |
| | 1 | 24488.3 | 0.18 | 24489.7 | -1.3 |
| | 2 | - | - | 24728.1 | - |
| L5C | 0 | 24192.9 | 0.16 | 24195.1 | -2.2 |
| | 1 | 24432.7 | 0.43 | 24433.5 | -0.9 |
| | 2 | 24670.9 | 0.95 | 24672.0 | -1.0 |
| E6C | 0 | 24178.0 | 0.22 | 24179.2 | -1.2 |
| | 1 | 24416.4 | 0.08 | 24417.6 | -1.2 |
| | 2 | 24654.7 | 0.11 | 24656.0 | -1.3 |
| I7C | 0 | 24194.2 | 0.03 | 24195.1 | -1.0 |
| | 1 | 24432.4 | 0.09 | 24433.5 | -1.1 |
| | 2 | - | - | 24672.0 | - |
| V99C | 0 | 24206.8 | 0.99 | 24209.2 | -2.3 |
| | 1 | 24446.5 | 0.11 | 24447.6 | -1.1 |
| | 2 | 24685.0 | 0.33 | 24686.0 | -1.0 |
| A101C | 0 | 24235.8 | 0.19 | 24237.2 | -1.4 |
| | 1 | 24474.7 | 0.15 | 24475.6 | -0.9 |
| | 2 | 24712.9 | 0.09 | 24714.0 | -1.2 |
| A103C | 0 | 24235.8 | 1.21 | 24237.2 | -1.4 |
| | 1 | 24474.4 | 0.54 | 24475.6 | -1.2 |
| | 2 | 24713.5 | 0.95 | 24714.0 | -0.5 |
| A106C | 0 | 24234.4 | 0.75 | 24237.2 | -2.9 |
| | 1 | 24474.3 | 0.36 | 24475.6 | -1.3 |
| | 2 | 24712.0 | 0.89 | 24714.0 | -2.0 |
| T111C | 0 | 24206.2 | 0.19 | 24207.2 | -1.0 |
| | 1 | 24444.1 | 0.35 | 24445.6 | -1.5 |
| | 2 | 24682.9 | 0.95 | 24684.0 | -1.1 |
| A112C | 0 | 24235.7 | 0.19 | 24237.2 | -1.5 |
| | 1 | 24474.5 | 0.27 | 24475.6 | -1.1 |
| | 2 | 24712.4 | 0.65 | 24714.0 | -1.6 |
| K113C | 0 | 24179.3 | 1.22 | 24180.1 | -0.9 |
| | 1 | 24416.2 | 0.82 | 24418.5 | -2.4 |
| | 2 | - | - | 24656.9 | - |
| A114C | 0 | 24236.3 | 0.16 | 24237.2 | -0.9 |
| | 1 | 24474.7 | 0.24 | 24475.6 | -0.9 |
| | 2 | 24712.1 | 0.64 | 24714.0 | -1.9 |
| K115C | 0 | 24179.5 | 0.67 | 24180.1 | -0.6 |
| | 1 | 24416.5 | 1.59 | 24418.5 | -2.1 |
| | 2 | - | - | 24656.9 | - |
| I116C | 0 | 24194.1 | 0.07 | 24195.1 | -1.0 |
| | 1 | 24433.1 | 0.21 | 24433.5 | -0.5 |

| | | | | | |
|---|---|---|---|---|---|
| | 2 | - | - | 24672.0 | - |
| I118C | 0 | 24194.0 | 0.19 | 24195.1 | -1.1 |
| | 1 | 24432.7 | 0.47 | 24433.5 | -0.9 |
| | 2 | - | - | 24672.0 | - |
| A178C | 0 | 24236.2 | 0.05 | 24237.2 | -1.0 |
| | 1 | 24474.4 | 0.65 | 24475.6 | -1.3 |
| | 2 | - | - | 24714.0 | - |
| L179C | 0 | 24193.5 | 0.26 | 24195.1 | -1.7 |
| | 1 | 24433.0 | 0.38 | 24433.5 | -0.5 |
| | 2 | - | - | 24672.0 | - |
| L180C | 0 | 24192.1 | 0.75 | 24195.1 | -3.0 |
| | 1 | - | - | 24433.5 | - |
| | 2 | - | - | 24672.0 | - |
| A181C | 0 | 24236.4 | 0.20 | 24237.2 | -0.9 |
| | 1 | 24474.9 | 0.92 | 24475.6 | -0.7 |
| | 2 | - | - | 24714.0 | - |
| A182C | 0 | 24236.0 | 0.53 | 24237.2 | -1.2 |
| | 1 | 24474.8 | 0.16 | 24475.6 | -0.9 |
| | 2 | 24712.2 | 0.81 | 24714.0 | -1.9 |
| S183C | 0 | 24219.4 | 0.77 | 24221.2 | -1.8 |
| | 1 | 24458.2 | 0.53 | 24459.6 | -1.5 |
| | 2 | 24697.3 | 0.85 | 24698.0 | -0.8 |
| A184C | 0 | 24235.1 | 0.20 | 24237.2 | -2.1 |
| | 1 | 24474.0 | 0.42 | 24475.6 | -1.7 |
| | 2 | 24712.1 | 1.08 | 24714.0 | -1.9 |
| P185C | 0 | 24210.7 | 0.76 | 24211.2 | -0.5 |
| | 1 | 24448.6 | 0.19 | 24449.6 | -1.0 |
| | 2 | 24686.4 | 0.42 | 24688.0 | -1.6 |
| P186C | 0 | 24210.1 | 0.12 | 24211.2 | -1.1 |
| | 1 | 24448.6 | 0.04 | 24449.6 | -1.0 |
| | 2 | 24686.3 | 0.41 | 24688.0 | -1.7 |
| **C-CPE** | | | | | |
| C-CPE | 0 | 14044.5 | 0.16 | 14045.7 | -1.2 |
| His-TEV-C-CPE | 0 | 16008.8 | 0.98 | 16008.8 | 0.0 |
| **Retinal bound Bacteriorhodopsin** | | | | | |
| wt | 0 | 31257.8 | 0.95 | 31257.4 | 0.4 |
| M32C | 0 | 31229.3 | 0.89 | 31229.4 | -0.1 |
| | 1 | 31467.7 | 1.17 | 31467.8 | -0.1 |
| A44C | 0 | 31289.7 | 0.88 | 31289.5 | 0.2 |
| | 1 | 31528.6 | 1.31 | 31527.9 | 0.7 |
| L109C | 0 | 31247.0 | 0.47 | 31247.4 | -0.4 |
| | 1 | 31486.8 | 0.25 | 31485.8 | 1.0 |
| F153C | 0 | 31213.2 | 0.84 | 31213.4 | -0.2 |
| | 1 | 31452.4 | 1.27 | 31451.8 | 0.6 |
| N176C | 0 | 31246.5 | 1.00 | 31246.5 | 0.0 |
| | 1 | 31484.6 | 1.26 | 31484.9 | -0.3 |
| R225C | 0 | 31205.5 | 1.37 | 31204.4 | 1.1 |
| | 1 | 31442.3 | 1.12 | 31442.8 | -0.5 |
| **KcsA** | | | | | |
| wt | 0 | 19513.1 | 0.65 | 19514.1 | -1.0 |
| S22C | 0 | 19528.6 | 0.71 | 19530.2 | -1.6 |
| | 1 | 19767.1 | 0.55 | 19768.6 | -1.5 |
| R27C | 0 | 19459.6 | 0.68 | 19461.1 | -1.5 |
| | 1 | 19698.2 | 0.84 | 19699.5 | -1.3 |

| CD20 | | | | | |
|---|---|---|---|---|---|
| wt | 0 | 37060.1 | 1.45 | 37061.5 | -1.4 |
| | 1 | 37299.7 | 1.15 | 37299.9 | -0.1 |
| | 2 | 37538.4 | 1.31 | 37538.3 | 0.1 |
| C111A | 0 | 37028.8 | 1.56 | 37029.4 | -0.6 |
| | 1 | 37268.0 | 0.87 | 37267.8 | 0.2 |
| C220A | 0 | 37028.2 | 1.15 | 37029.4 | -1.2 |
| | 1 | 37268.8 | 1.19 | 37267.8 | 0.9 |
| C111A,C220A | 0 | 36997.2 | 1.19 | 36997.3 | -0.1 |

**Supplementary Table 1.** Theoretical and measured masses of all measured (palmitoylated) proteins in this study.

| | | Model evaluation | | |
|---|---|---|---|---|
| Protein | Parameters | Independent | Cooperative | Cooperative model fit to all datasets |
| Cld3 | $p_{103}$ | 0.71 | 0.72 | 0.69 (0.65-0.72) |
| | $p_{106}$ | 0.72 | 0.72 | 0.69 (0.65-0.72) |
| | $p_{182}$ | 0.90 | 0.90 | 0.86 (0.83-0.90) |
| | $p_{184}$ | 0.64 | 0.64 | 0.64 (0.60-0.67) |
| | $p_{additional}$ | 0.10 | 0.08 | 0.10 (0.09-0.12) |
| | c | - | 1.31 | 1.24 (1.19-1.29) |
| CD20 | $p_{111}$ | 0.74 | 0.74 | 0.73 (0.69-0.77) |
| | $p_{220}$ | 0.82 | 0.82 | 0.81 (0.77-0.85) |
| | c | - | 1.24* | 1.51 (1.10-1.92) |

**Supplementary Table 2.** Parameters obtained from fitting the palmitoylation models to Cld3 and CD20 data sets. To evaluate the independent and cooperative models, parameters for Cld3 were obtained by fitting to Cld3 variants with a single cysteine at a native palmitoylation site and variants with all putative palmitoylated cysteines mutated to alanine. Parameters for CD20 were obtained by fitting both models to CD20 variants with a single cysteine at a native palmitoylation site. These parameters were used to predict distribution of palmitates on Cld3 variants with multiple palmitoylation sites and native CD20. For prediction of native CD20, the cooperativity parameter obtained from fitting the cooperative model to all Cld3 data sets (asterisk). Parameter values corresponding to 95 %-confidence intervals are shown between brackets of the fit of the cooperative model to all data obtained for Cld3 and CD20.

## Supplementary References


1. Suzuki, H. *et al.* Crystal structure of a claudin provides insight into the architecture of tight junctions. *Science* **344,** 304–307 (2014).
2. Shinoda, T. *et al.* Structural basis for disruption of claudin assembly in tight junctions by an enterotoxin. *Sci Rep* **6,** 33632 (2016).
3. Saitoh, Y. *et al.* Tight junctions. Structural insight into tight junction disassembly by Clostridium perfringens enterotoxin. *Science* **347,** 775–778 (2015).
4. Wu, J. *et al.* Structure of the voltage-gated calcium channel Cav1.1 complex. *Science* **350,** aad2395–aad2395 (2015).
5. Krissinel, E. & Henrick, K. Secondary-structure matching (SSM), a new tool for fast protein structure alignment in three dimensions. *Acta Crystallogr. D Biol. Crystallogr.* **60,** 2256–2268 (2004).
6. Sievers, F. *et al.* Fast, scalable generation of high-quality protein multiple sequence alignments using Clustal Omega. *Mol. Syst. Biol.* **7,** 539–539 (2011).
7. Luecke, H., Schobert, B., Richter, H. T., Cartailler, J. P. & Lanyi, J. K. Structure of bacteriorhodopsin at 1.55 A resolution. *J. Mol. Biol.* **291,** 899–911 (1999).
8. Zhou, Y., Morais-Cabral, J. H., Kaufman, A. & MacKinnon, R. Chemistry of ion coordination and hydration revealed by a K+ channel-Fab complex at 2.0 A resolution. *Nature* **414,** 43–48 (2001).
